# Generation of fusion protein EGFRvIII-HBcAg and its anti-tumor effect in vivo

**DOI:** 10.1186/1756-9966-28-133

**Published:** 2009-09-29

**Authors:** Xiao-yi Duan, Dong-gang Han, Ming-xin Zhang, Jian-sheng Wang

**Affiliations:** 1 Department of Nuclear Medcine, First affiliated Hospital of Xi'an Jiaotong University, Xi'an 710061, PR China; 2 Department of Oncology, First affiliated Hospital of Xi'an Jiaotong University, Xi'an 710061, PR China

## Abstract

The epidermal growth factor receptor variant III (EGFRvIII) is the most common variation of EGFR. Because it shows a high frequency in several different types of tumor and has not been detected in normal tissues, it is an ideal target for tumor specific therapy. In this study, we prepared EGFRvIII-HBcAg fusion protein. After immunization with fusion protein, HBcAg or PBS, the titers of antibody in BALB/c mice immunized with fusion protein reached 2.75 × 10^5^. Western blot analysis demonstrated that the fusion protein had specific antigenicity against anti-EGFRvIII antibody. Further observation showed fusion protein induced a high frequency of IFN-γ-secreting lymphocytes. CD4+T cells rather than CD8+T cells were associated with the production of IFN-γ. Using Renca-vIII(+) cell as specific stimulator, we observed remarkable cytotoxic activity in splenocytes from mice immunized with fusion protein. Mice were challenged with Renca-vIII(+) cells after five times immunization. In fusion protein group, three of ten mice failed to develop tumor and all survived at the end of the research. The weight of tumors in fusion protein were obviously lighter than that in other two groups (*t *= 4.73, *P *= 0.044;*t *= 6.89, *P *= 0.040). These findings demonstrated that EGFRvIII-HBcAg fusion protein triggered protective responses against tumor expressing EGFRvIII.

## Introduction

Epidermal growth factor receptor (EGFR) plays an important role in tumor cell proliferation, differentiation and survival. Increasing evidences suggest that alterations within the EGFR gene may be as important as EGFR-overexpression to induce oncogenic effects [1-3]. The most common variation is an in-frame deletion of exons 2-7 in the mRNA, resulting in a truncated mutant (epidermal growth factor receptor variant III, EGFRvIII). Even though EGFRvIII is lack of a portion of extracellular ligand-binding domain and can not bind to its ligand, the tyrosine kinase in the intracellular portion can be constitutively activated, thereby leading to receptor dimerization, autophosphorylation and stimulation of signal transduction cascades[4]. Because EGFRvIII is present with a high frequency in several different types of tumor and has not been detected in normal tissues, it is an ideal target for tumor specific therapy[5,6]. Among approaches directed to EGFRvIII, vaccine is a promising strategy.

Recombinant protein has been intensively studied as a vaccine on the basis of genetic engineering technology. Compared with peptide vaccine, recombinant protein has many advantages such as easy manipulation, mass production and low cost. The carrier of foreign epitope is important for construction of recombinant protein. Hepatitis B core protein (HBcAg) is one of the most promising delivery vehicles for its high-density, immunogenic presentation of foreign epitope and its production in various expression systems[7]. The e1 loop in the main determinant of the core antigen is considered as the most promising insertion site[8].

Pep-3, a 13-amino-acid peptide corresponding to the amino acid sequence of the EGFRvIII fusion junction (LEEKKGNYVVTDH), is an immunogenic peptide that was firstly reported by Moscatello[9]. In this study, foreign epitope, encoding Pep-3, was inserted into the immunodominant e1 loop of the HBcAg to prepare the recombinant fusion protein. Next, the antigenicity and immunogenicity of the fusion protein were detected in vitro. The protective immune responses against tumor was evaluated in a murine model.

## Materials and methods

### Construction of recombinant expression plasmids

The genes encoding Pep-3, HBcAg amino acid resides from 1 to 71 and from 89 to 144 were amplified by PCR, and a set of primers were listed in Table 1.

**Table 1:** Sequence of primers used for PCR amplification

**Gene**	**Primers sequence**	**Restriction site**
Pep-3	Sense: 5'-CCTCGAGCTGGAGGAGAAGAAAGGTAATTATGTG-3' Antisense: 5'-CGGATCCGTGATCTGTCACCACATAATTACC-3'	*Xho*l *Bam*HI
HBcAg_1-71_	Sense:5'-GGAATTCATGATTACGCCAAGCTTGGCTGCAGAGTTCCATATG-3' Antisense: 5'-CCCTCGAGTCCACCCCAGGTAGCTAGAGTC-3'	*EcoR*I *Xho*l
HBcAg_89-144_	Sense: 5'-CGGATCCGGTGGAGTCAACAACACTAATATGGGC-3' Antisense: 5'-CCAAGCCTTCCGCGGAAGTGTTGATAGGAT-3	*Bam*HI *Hind*III

**Figure 1:**
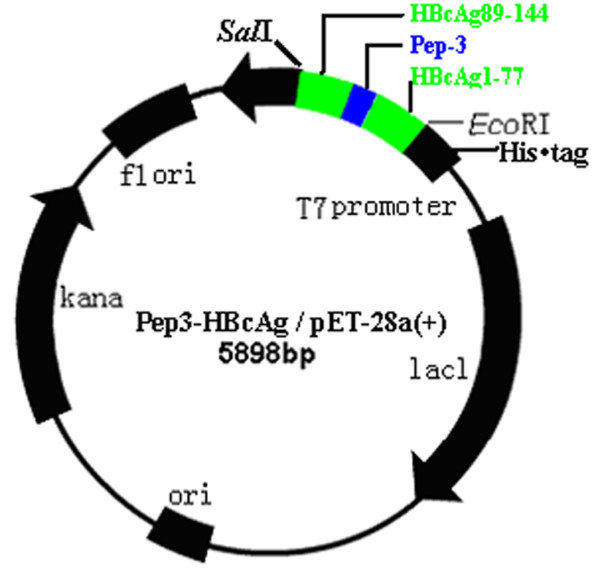
**Map of Pep3-HBcAg/pET-28a(+) prokaryotic expression plasmid**. The three DNA fragments were ligated and subcloned into plasmid pGEMEX-1. Then fusion gene Pep3-HBcAg was digested with restriction enzymes *Eco*RI and *Sal*I and ligated into the equivalent sites of the pET-28a(+) vector, yielding His-tagged Pep3-HBcAg/pET-28a(+).

Recombinant prokaryotic expression plasmid Pep3- HBcAg/pET-28a(+) was constructed(Figure 1), and then identified by restriction enzymes and sequencing.

### Expression and purification of the fusion protein in Escherichia coli

Recombinant plasmid Pep3-HBcAg/pET-28a(+) was introduced into *Escherichia coli *BL21 (DE3). Then isopropy-β-D-thiogalactoside (IPTG, Sigma) was added to induce fusion protein expression. The BL21 cells were harvested, supernatant and sediment were subjected for SDS-PAGE. As the fusion protein was confirmed to be present in inclusion bodies, a further lysis step was performed (8 M urea overnight). The supernatant was purified on a Ni^2+^-NTA affinity chromatography column (Novagen). The His-tag was removed and the concentration of purified fusion protein was measured with the Bradford assay. EGFRvIII-specific antibody (Zymed) was used to confirm the identity of the fusion protein.

### Immunization of mice and antibody detection

Thirty 6-8-week-old female BALB/c mice were purchased from Medical Experimental Animal Center, Xi'an Jiaotong University. All studies were performed in accordance with the Institutional Animal Care and Use Committee (IACUC) of Xi'an Jiaotong University. Ten mice were subcutaneously injected with fusion protein (100 μg/animal) emulsified in Freund's complete adjuvant (Sigma) on day 0 and with the same amount of protein emulsified in Freund's incomplete adjuvant on day 7. The third and following boosters were done only with fusion protein once a week with a total of seven immunizations. Other 20 mice were divided into two groups, and immunized with HBcAg and PBS. Immune serum samples were collected and stored at -70°C. Antibody titers were assayed by enzyme-linked immunosorbent assay (ELISA).

### IFN-γ detection

Enzyme-linked immunospot assay (ELISPOT) was used to evaluate tumor-specific IFN-γ-secretion in splenocytes. One week after the final vaccination, spleen cells from three mice per group were harvested. Immunospot plates were coated with 100 μl anti-mouse γ-IFN monoclonal antibody (5 μg/ml, BD PharMingen). Freshly isolated splenocytes were added into plate at a density of 3 × 10^6 ^cells/well and co-cultured with 1 μg/ml EGFRvIII-specific peptide (pep-3) for 20 h at 37°C. Medium without blood-serum was added as negative control. Plates were washed and incubated with 50 μl/well of biotin-conjugated anti-mouse IFN-γ, and then stayed overnight at 4°C. Then, 10 μl/well of HRP-labelled streptavidin was added. After extensive washing, 100 μl AEC substrate was added until development of visible spots. Plates were then washed, air-dried and spots were counted using an ELISPOT reader (CTL Co.). To reveal roles of CD4+and CD8+ T cells in the immune response, splenocytes were depleted of CD4+ or CD8+ T cells by using corresponding antibody (Miltenyi Biotec Inc.) before ELISPOT assays.

### Cytotoxicity assay

Splenocytes were harvested from three mice per group one week after the final vaccination, and then incubated with irradiated Renca-vIII(+)cells(EGFRvIII transfected Renca cells[10]). Five days later, T cells were harvested and purified from the cultures using lymphocyte separating buffer. These T cells were used as CTL effector cells and co-cultured with target cells renca-vIII(+)cells at various effector/target ratios for 8 h at 37°C. Values were expressed as the percentages of surviving Renca-vIII(+)cells cultured with effector cells. Renca cells which were not transfected with EGFRvIII served as control.

### Tumor challenge

Thirty BALB/c mice were divided into three group(10 mice pre group), and immunized with fusion protein, HBcAg and PBS. After five times of immunization, antibody titers of mice immunized with fusion protein reached 2 × 10^5^. Then all mice were challenged with 1.5 × 10^5 ^Renca-III(+) cells in the left flank. Tumor growth was measured and volumes were calculated according to the formula V = (a^2^·b^2^·c^2^)/6, where V represents tumor volume and a, b, and c were perpendicular diameters of the tumor. After observation, mice were killed, and tumors were weighted.

### Statistical analyses

All data were expressed as means ± SD. Comparisons between individual data points were performed by Student's *t*-test. Data for quantitation were evaluated by analysis of variance (ANOVA). *p *< 0.05 was considered statistically significant.

## Results

### Construction of recombinant expression plasmids

**Figure 2:**
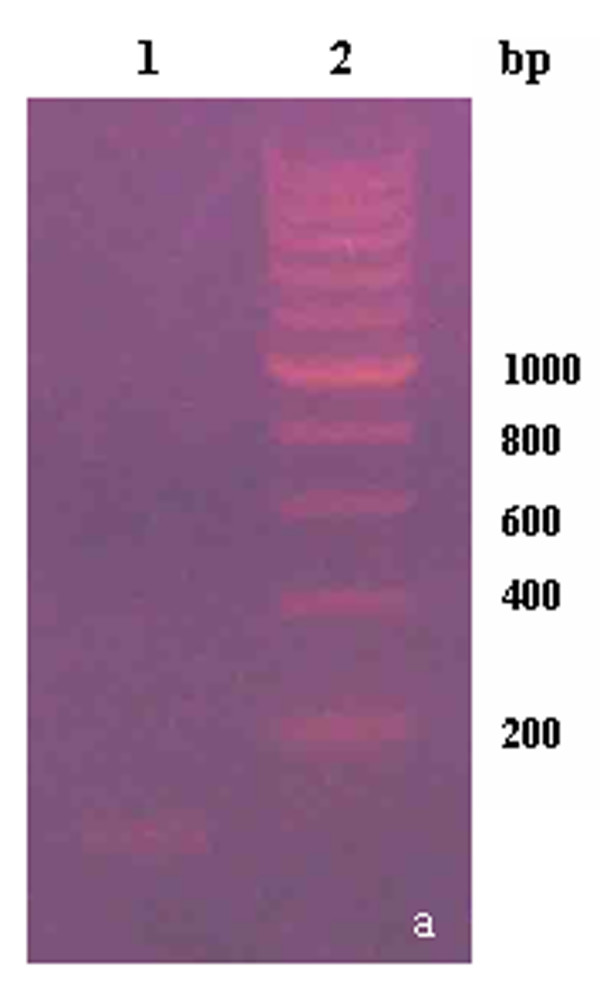
**Identification of PCR product**. lane1: PCR product of Pep-3; lane2: DNA Marker of 200 bp.

**Figure 3:**
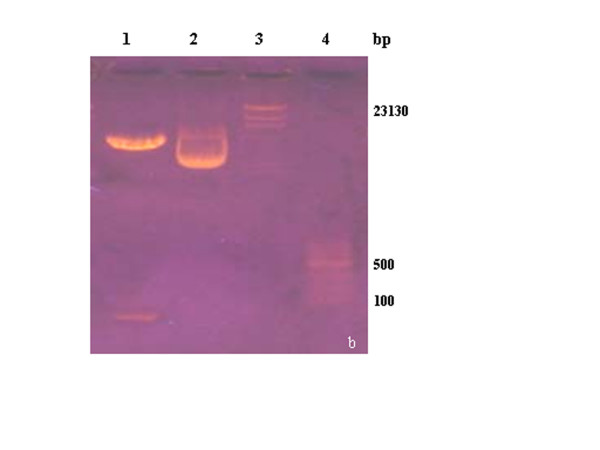
**Identification of plasmid Pep3-HBcAg/pGEMEX-1**. lane1: cloning plasmid Pep3-HBcAg/pGEMEX-1 digested with *EcoR *I and *Xho*I; lane 2: pep3-HBcAg/pGEMEX-1 plasmid without digestion; lane 3:λDNA/*Hind*III marker(23.13 Kb, 9.414 Kb, 6.557 Kb, 4.371 Kb, 2.082 Kb, 0.564 Kb, 0.125 Kb); lanel 4: 100 bp DNA Ladder.

**Figure 4:**
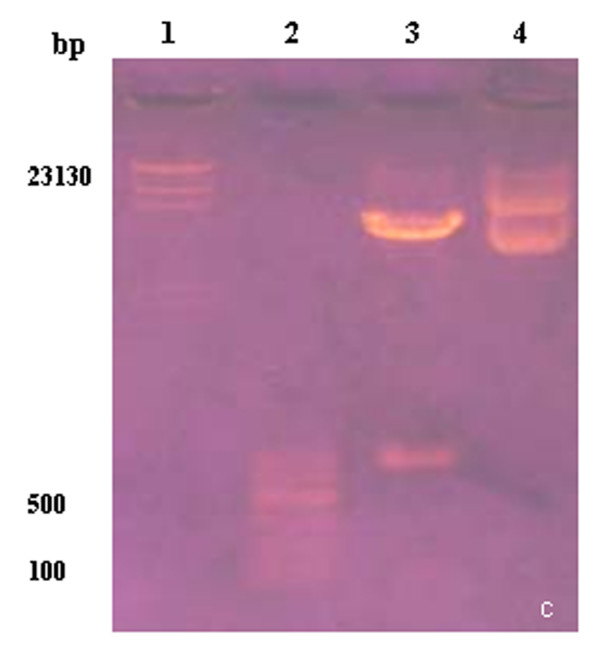
**Identification of plasmid pep3-HBcAg/pET-28a (+)**. Lanel1: λDNA/*Hind*III marker; lanel 2: 100 bp DNA Ladder; lane 3: recombinant expression plasmid pep3-HBcAg/pET-28a (+) digested with *EcoR *I and *Sal*I; lane 4: pep3-HBcAg/pET28a (+) plasmid without digestion.

The PCR product and recombinant plasmid were detected by restriction analysis (Figures 2, 3 and 4) and then sequenced. The results showed that the compound gene Pep-3, cloning plasmid Pep3-HBcAg/pGEMEX-1, and expression plasmid Pep3-HBcAg/pET-28a (+) were successfully constructed.

### Expression and purification of the fusion protein

**Figure 5:**
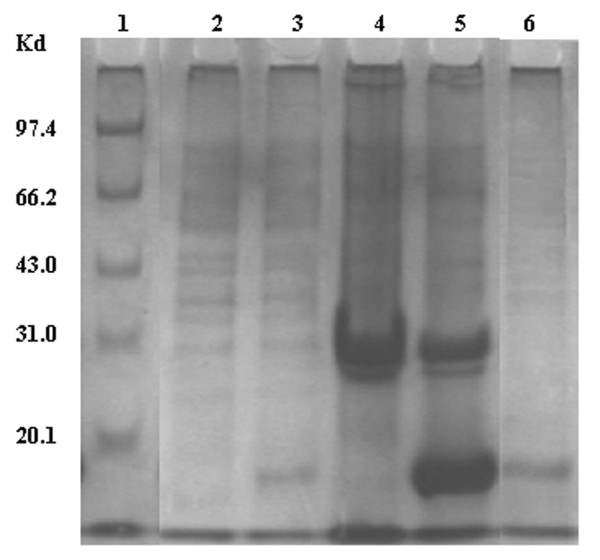
**SDS-PAGE analysis of recombinant protein**. Lane 1: protein molecular weight marker; Lane2: negative control: recombinant plasmid Pep3-HBcAg/pET28a (+) transformed *E. coli *BL21 cells not induced by IPTG; Lane 3: HBcAg/pET28a (+) transformed *E. coli *BL21 cells induced by IPTG Lane 4, 5: supernatant and sediment of recombinant plasmid Pep3-HBcAg/pET28a (+) induced by IPTG; Lane 6: purified recombinant fusion protein.

**Figure 6:**
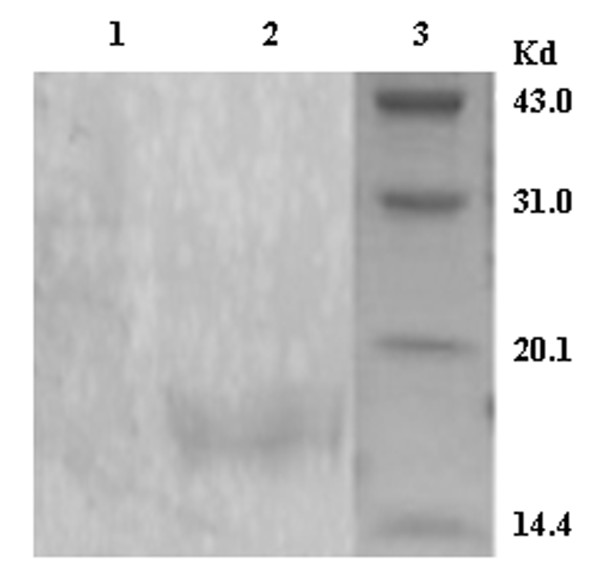
**Western blot analysis**. Lane 1: Western blot of pET28a (+); Lane 2: Western blot of EGFRvIII-HBcAg fusion protein using EGFRvIII-specific monoclonal antibody; Lane 3: protein marker.

To obtain the fusion protein, the engineering strains E. coli BL21 (DE3) were cultured in 2 × YT with 0.5% glucose and induced by IPTG at the concentration of 1 mM for 6 h. SDS-PAGE analysis demonstrated that recombinant fusion protein was efficiently and inducibly expressed in inclusion body form and could dissolve in 6 M urea. The molecular weight of the fusion protein was shown to be approximately 15.4 kD as expected. According to the results of SDS-PAGE and gel image analysis, the purified fusion protein accounted 92% of totle protein (Figure 5). Western blot analysis demonstrated that the fusion protein had specific antigenicity against anti-EGFRvIII antibody (Figure 6).

### Immunization assay of fusion protein

**Figure 7:**
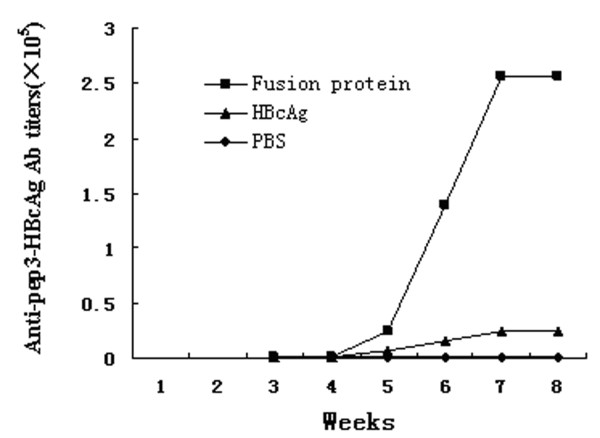
**Time course of immunization response**. Mice immunized with fusion protein produced specific antibody responses, which increased significantly from week 5 and peaked at week 7. However, no obvious antibody response was detected in mice immunized with HBcAg or PBS.

To investigate whether the EGFRvIII-HBcAg fusion protein could induce humoral immune response, the tail vein serum samples were collected on day 0, 14, 21, 28, 35, 42 and 48, and the antibody titers against the fusion protein were tested by ELISA (Figure 7).

### Induction of specific CTL response

**Figure 8:**
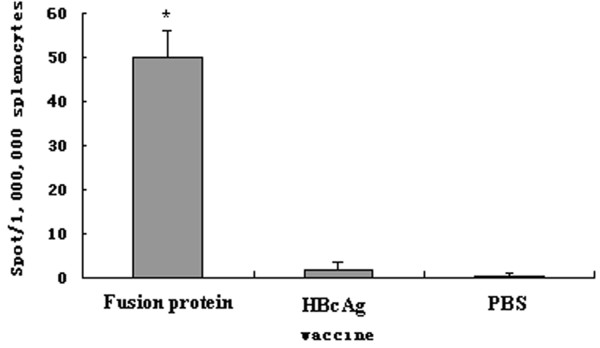
**Frenquency of IFN-γ-secreting cells in splenocytes from mice innunized with fusion protein was much higher than that in HBcAg or PBS**.

**Figure 9:**
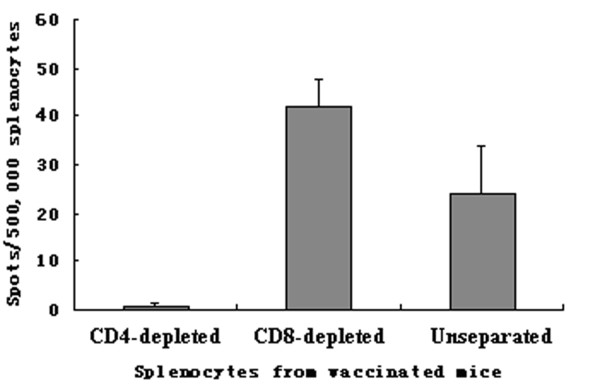
**Frequency of IFN-γ-secreting cells in CD4- depleted splenocytes was dramatically lower than CD8- depleted splenocytes**.

ELISPOT assay was carried out to determine the frequency of lymphocytes secreting IFN-**γ**. The number of IFN-**γ **secreting cells was very low in mice immunized with HBcAg or PBS alone, whereas vaccination with fusion protein induced a high frequency of IFN-**γ**-secreting cells (p < 0.05) (Figure 8). To identify which cell populations were involved in the IFN-**γ **production, the CD4- or CD8-depleted splenocytes from mice immunized with fusion protein were detected. The depletion of CD4+ T cells could completely abrogate IFN-**γ **production by the harvested splenocytes, but the depletion of CD8+T cells had no influence on the number of ELISPOTs (Figure 9). This finding suggest that CD4+ T cells, but not CD8+ T cells, play an important role in anti-tumor activity of fusion protein.

**Figure 10:**
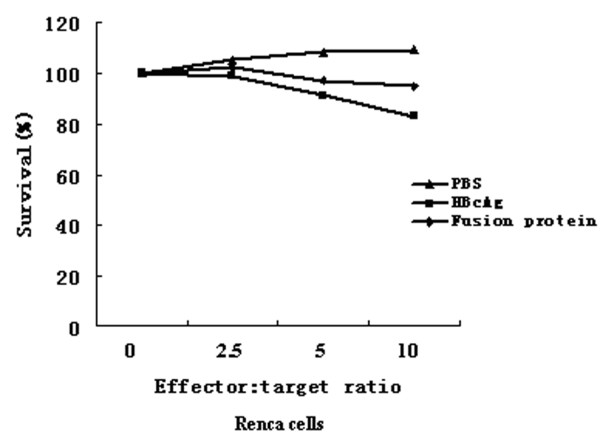
**Using Renca-vIII(+) cells as specific stimulator, cytolytic activity against Renca-vIII(+) cells was observed in splenocytes immunized with fusion protein**.

**Figure 11:**
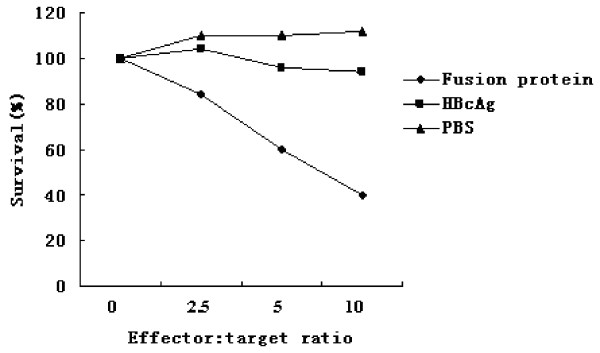
**Using Renca vIII(-) cells as specific stimulator cells**. cytolytic activity against Renca-vIII(+) cells was not obvious in splenocytes immunized with fusion protein.

Furthermore, the cytotoxic activity of splenocytes from mice was examined (Figure 10, 11). Using Renca-vIII(+) cells as specific stimulator in vitro, cytotoxic activity against Renca-vIII(+) cells was observed in splenocytes from mice immunized with fusion protein, whereas there was no obvious killing activity in splenocytes from mice that received HBcAg or PBS (p < 0.001). Using Renca cells without EGFRvIII transfection as stimulator, no obvious cytotoxic activity was observed in the three groups.

### Protective antitumor activity

**Figure 12:**
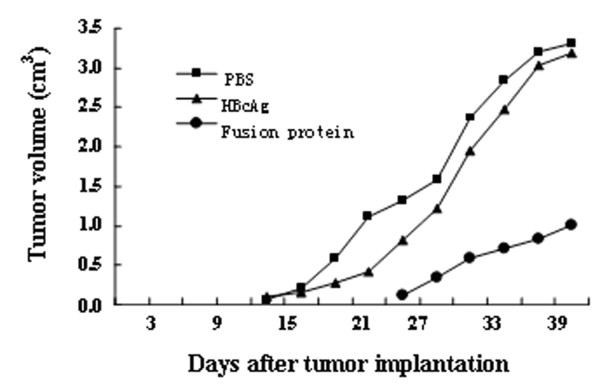
**Tumor growth curve of BALB/c mice immunized with fusion protein, HBcAg or PBS**. Among the three groups, immunized with fusion protein showed resistance to tumor development.

**Figure 13:**
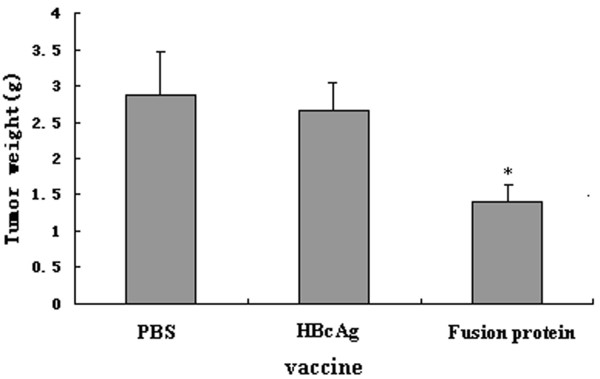
**Comparison of mean weight of tumor, mice immunized with fusion protein, the mean weight of tumors was significantly less than that in HBcAg or PBS group (p < 0.05)**.

Mice were challenged with Renca-vIII(+) cells after immunization for five times, and the tumor progression was observed. By day 21 after tumor implantation, all mice in HBcAg and PBS groups developed significant tumors. In mice immunized with fusion protein, three of ten mice failed to develop tumor and all survived at the end of the research. The mean size and weight of tumors in each group were monitored every three days. Results were shown in Figure 12 and 13. These results demonstrated that immunization with EGFRvIII-HBcAg fusion protein resulted in protective effect against tumor.

## Discussions

A major obstacle for efficient antitumor therapy is lack of specificity. The variant EGF receptor, EGFRvIII, is tumor specific, and is correlated with tumor progression and poor survival [11-14]. It has been reported that Pep-3 peptide can generate EGFRvIII-specific antitumor immune responses [15-18]. So, EGFRvIII is a potential therapeutic target. Genetically engineering vaccine is simple and relatively inexpensive to prepare in large quantities. In this study, we designed recombinant plasmids with insertion of EGFRvIII Pep-3 epitope into the immunodominant e1 loop of the HBcAg and observed adequate expression of recombinant fusion proteins in E. coli. This fusion protein could selectively combine with EGFRvIII-specific antibody, which showed fusion of HBcAg and EGFRvIII epitopes did not affect the antigenicity of EGFRvIII sequence.

Using ELISA, we found that the titers of anti-fusion protein antibody in mice immunized with fusion protein were much higher than that in HBcAg or PBS group. We further observed that fusion protein resulted in a high frequency of IFN-γ-secreting lymphocytes, which suggests that the IFN-γ response is tumor-specific and Th1-type dominant immune response. Next analysis showed CD4+T cells rather than CD8+T cells were associated with the production of IFN-γ. Using Renca-vIII(+) cell as specific stimulator, we observed remarkable cytotoxic activity in splenocytes from mice immunized with fusion protein, which further indicates that anti-tumor effect of fusion protein is EGFRvIII-specific. In vivo study, immunization with fusion protein can better protect mice from EGFRvIII(+) tumor cell challenge.

It has been confirmed that CD4+ and CD8+ T lymphocytes play important roles in induction of anti-tumor immune. In this study, EGFRvIII-HBcAg fusion protein induced antitumor immunity, and this immunity was mainly mediated by CD4+ T cells. There are two possible explanations for the effect mechanism of CD4+ T lymphocytes. One is the requirement of CD4+ T cells for the induction of natural killer cells and inhibition of tumor through IFN-γ production by T cells and IFN-γ receptor expression[19,20]. Another possible explanation is CD4+ T cell-mediated antibody production[21]. Patel D tested the anti-EGFR monoclonal antibody cetuximab for its interaction with EGFRvIII, and he found cetuximab could bind specifically to the EGFRvIII on the cell surface, thus leading to at least 50% of the cetuximab-EGFRvIII complex internalized from cell surface. This internalization led to a reduction in phosphorylated EGFRvIII in transfected cells, thus resulting in 40-50% inhibition of cell proliferation[22]. So, we presume that EGFRvIII-HBcAg fusion protein induces mainly humoral response and produces antigen-specific antibodies. The antibodies combined with EGFRvIII on the surface of tumor cells may result in receptor down-regulation and block tyrosine kinase activity, which inhibit the growth of tumor or protect body against EGFRvIII(+) tumor challenge.

In summary, we successfully prepared the EGFRvIII-HBcAg fusion protein. Immunization of animals with fusion protein stimulates an Ag-specific humoral response, and confers protective immunity to tumor challenge of EGFRvIII(+) tumor cells. We hope our approach will be helpful to the further research into a viable practical tumor vaccine.

## Competing interests

The authors declare that they have no competing interests.

## Authors' contributions

Xiao-yi Duan carried out the molecular genetic studies, participated in the sequence alignment and drafted the manuscript. Dong-gang Han carried out the immunoassays and participated in the sequence alignment. Ming-xin Zhang participated in the design of the study and performed the statistical analysis. Jian-sheng Wang conceived of the study, and participated in its design and coordination. All authors read and approved the final manuscript.
